# The Book World of Medicine and Science

**Published:** 1897-06-19

**Authors:** 


### The Book World of Medicine and Science.

Genito-Urinary Surgery and Venereal Diseases. By J. William White, M.D., and Edward Martin, M.D. Illustrated with 243 engravings and seven coloured plates. (London: J. B. Lippincott Co. 1897. 1,061 pp.)

It would be difficult to find a book illustrating better than this does the artificiality of the divisions according to which specialists divide the body between them. Truly the genito-urinary organs would seem to form an anatomical area capable of fairly distinct definition. But no sooner does one come to deal with its diseases than we find that they entirely decline to confine themselves to the region indicated, so that the medical man who would treat them must perforce bring his powers to bear upon a whole multitude of ailments affecting in some cases, as for example in syphilis, almost every part of the frame. While, moreover, a specialism may spread its net so as to include much that seems at first sight to be far outside its bounds, those who practise it are found to impose narrow and apparently arbitrary limits upon its extension in certain directions. In this book, for example, while in regard to syphilis, affections of the skin, the brain, the bones, the spleen, &c., are all included, a strict limitation is placed upon genito-urinary diseases occurring in women, none being treated of except such as have a venereal origin. The book is, in fact, a work on the genito-urinary diseases of man, and the venereal diseases of woman. It must be said at once that on its own subject as thus defined it is an admirable book, full of careful clinical description, and of practical hints in regard to treatment. In the treatment of gonorrhœa it is pointed out that copious irrigation of the urethra with dilute antiseptic solutions is often attended with a much more rapid subsidence of the inflammatory symptoms than is found to occur with the more ordinary methods. A quart irrigating bag or bottle is used and a small soft rubber catheter fitted to the nozzle is carried back to as far as the compressor urethræ muscle. Weak potassium permanganate seems to be the most popular injection; or perchloride of mercury, 1 in 20,000, may be used, a drachm of chloride of sodium being added to every pint, by which its specific gravity is raised, and it is rendered less irritating. In the section on the treatment of stricture of the urethra, the importance of thoroughly cleansing the meatus and its neighbourhood before passing any instrument is insisted on, and it is pointed out how especially necessary this is before passing a catheter in women; "hence catheterisation by touch alone is to be discouraged." There can, in our opinion, be no doubt about the importance of this advice. The old teaching that the female catheter should be passed under the bedclothes is undoubtedly responsible for much cystitis. Very careful directions are given as to the care of urethral instruments, the instructions as to their storage and sterilisation being of a somewhat elaborate nature. The vapour of formol is especially recommended for soft instruments. Needless to say all these precautions are rendered of no avail if the urethral opening through which the instruments pass is left in a septic condition. The chapter on syphilis and its various manifestations is very complete, and the illustrations are admirable, as in fact is the case throughout the book, some of the photographs of tertiary syphilides and gummatous ulceration being particularly good, and it may be added particularly horrible. Altogether the book is one which may be read with great satisfaction. Not only is it full of information, but this is so conveyed as to inspire confidence that it is not a compilation, but is the outcome of a large practical experience in the management of the class of diseases dealt with, of which indeed the names of the authors should be a sufficient guarantee.

The Municipal Year Book for 1897. By Robert Donald. (London: Offices of "London." Price 2s.)

This is a small handbook full of information in regard to the constructive work which is being carried on by our various municipalities. One of the most striking features of modern municipal life is the extent to which the corporations have entered into what may be called remunerative undertakings. These municipal industries include the supply of water, gas, and electricity, the management of tramways, and the erection of artizans' dwellings and municipal lodging-houses. These are all described in the book before us, separate sections being devoted to each topic. London is first treated by itself. Then comes a sketch of municipal government in England, after which there is a description of the government of the towns of England, Scotland, and Ireland. A good deal of information is squeezed into small compass in regard to all these towns, and it is especially to be noted that the names of the councils and of the chief officials are in all cases given. This is a most useful feature.

The London Health Laws, with Special Reference to the Dwellings of the Poor. Issued by the Mansion House Council on the Dwellings of the Poor. (London: Cassell and Co. 1897.)

This is a book which has already gone through four editions, and it is thus not necessary to say much about it beyond the fact that it appears to have been thoroughly brought up to date. The motto which the Mansion House Council ought to take for itself should surely be, *Quis custodiet ipsos custodes*, for the aim and object of its existence seems to be to keep the vestries and other representative bodies in order and force them to do their duty. It is strange that the people of London should choose year by year to appoint rulers over them who require such an organisation to compel them to do the work which is laid upon them by statute; yet such appears to be the case, and while such a condition of affairs continues it is clearly most desirable that every citizen should be able to find out easily and with certainty what the vestries are bound to do for the preservation of public health so as to know when and under what circumstances he may call them over the coals. For this purpose the first part of the book is admirably suited, dealing as it does with the law as to public health, buildings, and water supply. The second part deals with the Housing of the Working Classes Act, the third with Clearances by Private Enterprise, and an appendix contains many forms and definitions which may usefully be studied before any action is taken by those who are litigiously inclined. We have carefully gone through the book, which seems very correct so far as it goes.

